# Identifying non-accidental fractures in children aged <2 years

**DOI:** 10.1007/s11832-016-0755-3

**Published:** 2016-06-23

**Authors:** Laura A. Leaman, William L. Hennrikus, James J. Bresnahan

**Affiliations:** 1 Department of Orthopaedics and Rehabilitation, The Pennsylvania State University College of Medicine, Hershey, PA USA; 2 Department of Family Medicine, Lancaster General Health, Lancaster, PA USA; 3 Pennsylvania State University College of Medicine, 500 University Dr., Hershey, PA United States

**Keywords:** Child abuse, Trauma, NAT, Fracture, Non-accidental

## Abstract

**Purpose:**

Fractures are the second most common presentation of child abuse following soft-tissue bruising and burns. It is often difficult to determine potential abuse in a child presenting with a non-rib fracture(s) and without soft-tissue injuries.

**Methods:**

One hundred and fifteen consecutive patients aged ≤2 years who presented with a fracture between January 2010 and June 2012 to our emergency department (ED) or pediatric fracture clinic were retrospectively analyzed. Statistical analyses were carried out for non-accidental fractures based on age (<1 year vs 1–2 years), location of presentation (ED vs pediatric fracture clinic), type of long bone fracture, number of fractures, and patient demographics.

**Results:**

Fractures in 19 of 115 (17 %) patients were reported as non-accidental trauma (NAT). Eighty (70 %) of the 115 patients first reported to the ED. Thirty-two percent of fractures in children aged <1 year and 5 % of fractures in children aged 1–2 years were reported as NAT (*p* < 0.001). Sixteen of 19 (84 %) patients reported for abuse had multiple fractures; 15 of these patients were aged <1 year. Eight of 11 (73 %) reported femoral fractures were transverse fractures. Corner fractures (12) only occurred in children aged <1 year and never occurred in isolation; all of them were reported as NAT. Four of 60 patients (7 %) with commercial insurance and 15 of 55 patients (28 %) with Medicaid were reported as NAT.

**Conclusions:**

Age less than 1 year, multiple fractures, corner fractures, transverse fractures, and covered by Medicaid were the most common factors associated with reporting of NAT.

## Introduction

Fractures are the second most common presentation of child abuse following soft-tissue bruising and burns [1–8]. Previous studies have demonstrated that the majority of abuse-related fractures occur in children aged <1 year [2, 3, 5, 9–15]. In addition, these studies have established rib fractures in young children as highly indicative of abuse [4, 6, 7, 10, 11, 13–19]. Children aged <1 year are defenseless, non-ambulatory, and unable to communicate [3, 5, 6]. As such, it may be challenging to determine potential abuse in a child presenting with a single non-rib extremity fracture. This study aims to compare non-rib fracture presentation, the rate at which they are reported, characterization of the fractures suspicious for abuse, and patient demographics, in relation to age.

## Patients and methods

The College of Medicine Institutional Review Board (IRB) approved this study. A retrospective chart review was performed on 115 consecutive patients aged <2 years who had sustained at least one fracture between January 2010 and June 2012. Our database consisted of all pediatric fractures excluding skull fractures and rib fractures reported through the Pediatric Emergency Department (ED) and the Pediatric Bone and Joint Clinic at the College of Medicine. Inclusion criteria included any child aged <2 years who presented to either site with a fracture during the specific time frame. Data obtained from these charts included age, gender, race, insurance, mechanism of injury, number and description of fractures, additional injuries, skeletal survey results, and classification of possible abuse. If the child presented with a long bone fracture and the skeletal survey showed a rib or skull fracture, the study was included, but the rib or skull fracture was not included in the data.

All cases that were submitted to the Pennsylvania Office of Children, Youth, and Families for review were considered cases of potential abuse. The decision to report the child for non-accidental trauma (NAT) evaluation was made by the pediatric emergency attending physician or the orthopedic surgeon caring for the child. The ED physician was the reporting physician in 17 of 19 cases and the orthopedic surgeon was the reporting physician in two cases from the clinic. All cases reported were done so on the basis of clinical intuition, inconsistent histories, and fracture patterns. Reporting was consistent with the American Academy of Pediatrics (AAP) clinical guidance report [20]. Due to medical-legal barriers, we do not have information regarding substantiation by the Pennsylvania Office of Children, Youth, and Families.

Patients were divided by age into two groups—<1 year and 1–2 years. These groups were analyzed by race, insurance, fracture type, and classification of abuse. Descriptive statistics were utilized. Comparisons between age groups were performed using chi-squared test for non-accidental fractures. A *p* value of <0.05 was considered significant.

## Results

Of the 115 patients, 59 were female (51 %), and 56 were male (49 %). Fifty patients were aged <1 year and 65 were aged between 1 and 2 years. The patient population was 70 % White, 6 % African American, 7 % Hispanic, 7 % other, and 6 % unknown, as determined by the documented race in each patient’s medical record. This race distribution is comparable with Pennsylvania demographics in 2012, which indicate 79 % White, 11 % African American, 6 % Hispanic, and 5 % other [21].

**Fig. 1:**
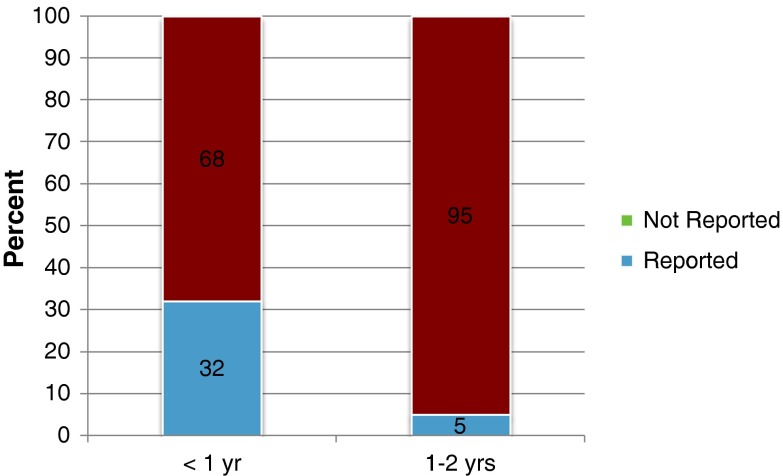
Overall distribution of fractures in relation to age. Thirty-two percent of those aged <1 year who presented with fractures were reported as NAT as opposed to only 5 % of those aged 1–2 years

Nineteen of 115 patients (17 %) were reported as abuse and 96 (83 %) were considered accidental injuries. Sixteen of 50 (32 %) children aged <1 year with fractures were reported as abuse, while three of 65 (5 %) children aged 1–2 years with fractures were reported as abuse (*p* < 0.001) (Fig. 1). Ten percent of fractures in males and 23 % of fractures in females (*p* = 0.059) were reported for abuse (Table 1).

**Table 1:** Abuse classification as related to age and gender

Abuse classification	<1 year (%)		1–2 years (%)		Total (%)	
Gender	M	F	M	F	M	F
Not reported	14 (70)	20 (66)	39 (100)	23 (89)	53 (90)	43 (77)
Reported	6 (30)	10 (33)	0 (0)	3 (11)	6 (10)	13 (23)
Total	20 (100)	30 (100)	39 (100)	26 (100)	59 (100)	56 (100)

Males and females were equally likely to be reported for NAT (*p* = 0.059)

### Multiple fractures

For each of the 115 patients, the number, location, and type of fracture were analyzed. Forty-seven (41 %) of the patients sustained multiple fractures (Table 2). Twenty-three patients with multiple fractures were aged <1 year and 24 were aged between 1 and 2 years. Sixteen of the 47 (34 %) patients with multiple fractures were reported for abuse, which constituted 84 % of the patients suspicious for abuse. In those reported for abuse, 13 of the 16 (81 %) patients with multiple fractures were aged <1 year, while three (19 %) were aged 1–2 years. Children presenting with multiple fractures not reported for abuse had fractures resulting from motor vehicle accidents, a lawn mower accident, and witnessed falls that resulted in combination radius/ulna or tibia/fibula fractures. Three of 19 (16 %) patients reported for abuse had a single fracture. The remainder of each child’s skeletal survey was normal. These included a femur fracture in a 2-month-old female, and a clavicle fracture in both a 2-month-old female and a 7-month-old female. The clavicle fracture in the 2-month-old female occurred after birth.

**Table 2:** Multiple fractures as related to abuse classification

Abuse classification	<1 year (%)		1–2 years (%)		Combined age groups	
Fractures	Single	Multiple	Single	Multiple	Single	Multiple
Not reported	24 (89)	10 (43)	41 (100)	21 (88)	65 (96)	31 (66)
Reported	3 (11)	13 (57)	0 (0)	3 (12)	3 (4)	16 (44)
Total	27 (100)	23 (100)	41 (100)	24 (100)	68 (100)	47 (100)

Forty-one percent of all patients had multiple fractures and 57 % of those aged <1 year with multiple fractures were reported as NAT

### Long bone fractures

Long bone fractures are defined as humerus, femur, or tibia. Sixty-two patients (55 %) presented with a long bone shaft fracture. Thirteen (21 %) of 62 patients with long bone shaft fractures were reported as abuse. In children aged <1 year, 11 of 33 (33 %) long bone fractures were reported. In children aged 1–2 years, two of 29 (7 %) with long bone fractures were reported as abuse (*p* = 0.061).

### Shaft fractures

Eight of 11 (73 %) humeral shaft fractures in children aged <1 year and two of 16 (12 %) humeral shaft fractures in children aged 1–2 years were reported for abuse (*p* = 0.011). Twelve patients sustained supracondylar fractures, but none were reported for abuse. There were two transphyseal fractures—one in a 4-month-old male who was found to have 18 additional fractures by skeletal survey, and the other in a 3-month-old child with a humeral shaft and rib fracture in addition to bilateral retinal and cerebral hemorrhages. Both of these cases were reported for abuse.

Eleven of 24 (46 %) femoral shaft fractures in children aged <1 year were reported for abuse. Femoral shaft fractures consisted of eight transverse and three spiral fractures. None of five (0 %) femoral fractures in children aged 1–2 years were reported for abuse (*p* = 0.297).

**Fig. 2:**
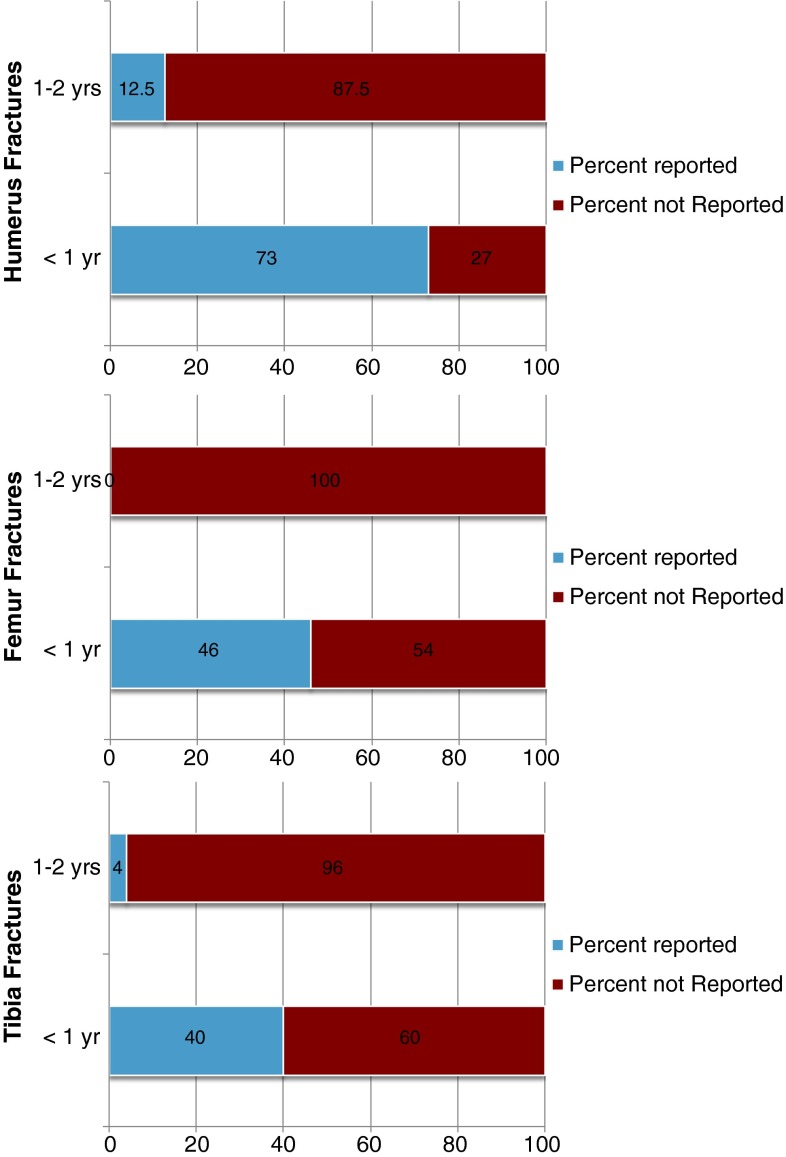
Reported and not reported fractures by location and age. Humerus factures in children aged <1 year were the most common fractures reported as NAT. Children aged 1–2 years with long bone fractures were not commonly reported as NAT

Six of 15 (40 %) tibia shaft fractures in children aged <1 year were reported for abuse. Only one of 22 (4 %) tibia shaft fractures in children aged 1−2 years (*p* = 0.042) were reported for abuse (Table 3; Fig. 2).

**Table 3:** Distribution of long bone fractures among age groups

Fracture location	Abuse	<1 year (%)	1–2 years (%)	Total (%)
Humerus	Not reported	3 (27)	14 (88)	17 (63)
	Reported	8 (73)	2 (12)	10 (37)
	Total	11 (100)	16 (100)	27 (100)

		Shaft	Corner	Shaft	Corner	
Femur	Not reported	13 (54)	0 (0)	5 (100)	0 (0)	18 (51)
	Reported	11 (46)	6 (100)	0 (0)	0 (0)	17 (49)
	Total	24 (100)	6 (100)	5 (100)	0 (0)	35 (100)
Tibia	Not reported	9 (60)	0 (0)	21 (95)	0 (0)	30 (70)
	Reported	6 (40)	6 (100)	1 (5)	0 (0)	13 (30)
	Total	15 (100)	6 (100)	22 (100)	0 (0)	43 (100)

Six of 15 tibia shaft fractures were reported, and six tibial corner fractures were reported

### Corner fractures

Six of 30 femur fractures and six of 21 tibia fractures were corner fractures. The average age of patients with corner fractures was 2.2 months, ranging from 1 month to 4 months. All corner fractures in this study were reported as abuse, comprising 35 % of femur fractures, and 46 % of tibia fractures. No corner fractures of the humerus occurred. No corner fractures occurred in isolation.

### Location of initial presentation

Thirty-five of 115 patients (30 %) with a fracture(s) were initially evaluated in the pediatric orthopedic clinic, while 80 patients (70 %) were first seen in the ED. Of children aged <1 year, 13 (26 %) presented to the clinic and 37 (74 %) presented to the ED. In children aged 1−2 years with fractures, 22 (34 %) first presented to the clinic and 43 (66 %) first presented to the ED.

**Fig. 3:**
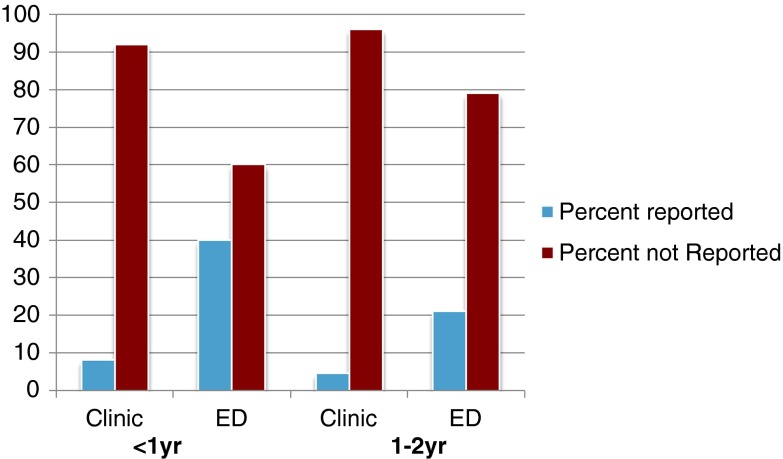
Reported and not reported fractures by age and initial location of presentation. Forty percent of those aged <1 year who presented to the ED were reported as abuse

Children reported for abuse who were aged <1 year were more likely to initially present to the ED than to the clinic (*p* < 0.001). Similarly, children reported for abuse aged 1–2 years were more likely to report to the ED than clinic (Fig. 3).

In general, more severe injuries presented to the ED. For example, 33 of 35 (94 %) femoral shaft fractures, 28 of 43 (65 %) tibial shaft fractures, and 22 of 27(82 %) humeral shaft fractures presented to the ED. Children who were reported for abuse, regardless of age, were more likely to present to the ED (21 %) compared to the clinic (6 %). Those aged <1 year and reported for abuse were eight times more likely to present to the ED (40 %) compared with their 1–2-year-old counterparts (5 %) (*p* < 0.001). Of the 19 patients reported as abuse, 17 (90 %) were reported in the ED. In only two of 19 cases (10 %) was the orthopedic surgeon the first doctor to make the report.

Location of presentation was further analyzed by fracture type (Table 4). Five of 27 (19 %) humerus fractures, and two of 35 (6 %) femur fractures presented to clinic, none of which were reported for abuse. Fifteen of 43 (35 %) tibia fractures presented to the clinic. Two of the 15 tibia fractures that reported to the clinic (13 %) were also reported for abuse. These two tibia fractures were reported as NAT by the orthopedic surgeon in the clinic.

**Table 4:** Distribution of reported NAT by location of presentation and fracture type

Fracture	Total reported	ED reported	Clinic reported
Humerus	27 (10)	22 (10)	5 (0)
Femur	35 (17)	33 (17)	2 (0)
Tibia	43 (13)	28 (10)	15 (2)

Nearly every fracture reported was first evaluated in the ED. Three of 40 fractures reported as NAT first presented to an orthopedist

### Additional injuries

Sixteen of 115 patients (14 %) sustained additional injuries including bruising, lacerations, and intracranial hematomas, of which half (8 of 16) were reported for abuse. The eight cases not reported for abuse resulted from motor vehicle accidents, a trampoline accident, and a lawn mower accident.

### Insurance coverage

Four of 60 (7 %) patients with commercial insurance were reported for abuse compared to 15 of 55 (28 %) of patients with Medicaid.

### Race

Overall, percentages of reported abuse were highest in African American (43 %), unknown (29 %), and Hispanic patients (25 %), followed by White (12 %) and other (12 %).

## Discussion

The purpose of this study is to compare the spectrum of non-accidental fractures in children aged <1 year with those aged 1–2 years. The current study found age <1 year, multiple fractures, corner fractures, transverse fractures, and having Medicaid insurance were the most common factors associated with reporting of NAT.

When infants and toddlers present with a fracture in the absence of a known cause, physical abuse should be considered. Kemp et al. reported that the high number of quality research studies in this field is limited [12].

An abused child who returns to an unsafe home environment is at risk for additional injury [1–3, 5, 6, 22]. In cases where ‘reasonable suspicion’ is difficult to determine, consultation with a child abuse pediatrician may be helpful [4]. As outlined by the AAP Committee on Child Abuse and Neglect, this evaluation may include interviewing multiple family members, speaking with the child’s primary care physician, comparing the proposed mechanism of injury with the fracture itself, and further physical examination for additional injuries, including head and neck or sexual injuries [20].

Multiple fractures in a young child, without a causal mechanism such as a motor vehicle accident, are highly suspicious for abuse. Sources have reported 70–83 % of abused children aged <1 year had at least two fractures [6, 13, 19]. We found that 84 % (16 of 19) of cases of multiple fractures were reported for potential abuse and 79 % of all reported cases were in children aged <1 year who also had multiple fractures.

A few authors suggest that a single long bone diaphyseal fracture is the most common fracture pattern identified in abused children [4, 23, 24]. The results of the current study disagree with this notion. Only three of 19 cases of reported abuse were single long bone fractures. The current study agrees with the findings of Kocher and Kassen, Leventhal et al. and Worlock et al. who demonstrated that multiple fractures are the most common presenting pattern of non-accidental fractures [6, 13, 19].

In the three reported cases where there was only a single fracture, each case was considered suspicious for abuse due to a variety of factors highlighted by the AAP [20]. Physical examination findings consistent with abuse include any injury to a young perambulatory infant, injuries to multiple organ systems, multiple injuries in different stages of healing, patterned injuries, injuries to non-bony or other unusual locations, significant unexplained injuries, and additional evidence of neglect [20].

Studies have investigated the correlation between long bone fractures and abuse in children aged ≤2 years [5, 22, 23, 25]. Previous studies have shown a higher rate of abuse in children aged <1 year compared to their 1–2-year-old counterparts [25–27]. Our results agree with these previous studies. The overall rate of cases reported for abuse in children aged <2 years was 17 %. When analyzed by age group, 32 % of children aged <1 year compared to 5 % of children aged 1–2 years presented with fractures that were reported for abuse (*p* < 0.0001). Some historical studies have reported abuse rates ranging between 50 and 69 % in children aged <1 year presenting with fractures [2, 6, 7, 9, 24]. However, more recent studies of fractures in children aged <1 year report rates of ~25 % [4, 14, 15, 26].

Multiple studies have identified that humeral shaft fractures in children aged <1 year are strongly suggestive of abuse, with reported rates between 36 and 100 % [2, 25, 26]. In the current study, eight of 11 (73 %) humeral shaft fractures in patients aged <1 year compared to two of 16 (12 %) humeral shaft fractures for children aged 1−2 years were reported as abuse.

Previous authors have indicated that only 0.5 % of supracondylar humerus fractures are reported as abuse [4, 6, 12, 13, 25, 28, 29]. The current study also supports this finding as no patients with supracondylar fractures were reported as abuse.

Femoral shaft fractures in non-ambulatory children are highly suspicious for abuse and are reported as such in 17–80 % of cases [1, 4–7, 12, 22, 23, 25, 29]. In the present study, 11 of 24 (46 %) femoral shaft fractures in children aged <1 year were reported for abuse while none of the five femoral shaft fractures in children aged 1−2 years were reported. Our findings support the idea that children aged ≥1 year with a femoral shaft fracture are more likely to be reported as abuse (*p* = 0.011). Non-ambulatory infants are typically unable to generate the force required to produce an accidental femur fracture whereas ambulatory toddlers, fearless in exploring their environment, are able to generate this force. Pennock et al. [30] demonstrated that falls by a carer while carrying a small child downstairs can result in accidental femur fractures.

Spiral femur fractures were once thought to be the most common fracture type observed in abuse. More recent studies suggest transverse fractures are the most common fracture type among children with non-accidental fractures, which is consistent with the findings in this study [20, 22]. Eight of 11 femoral shaft fractures suspicious for abuse were transverse fractures.

Tibial shaft fractures are the third most commonly fractured long bone in children following the humerus and femur [25, 31]. However, tibia fractures are cited less often in the literature in association with child abuse, especially in a non-ambulatory child [13, 20, 26]. In the present study, six of 15 (40 %) tibia fractures in children aged <1 year compared to one of 22 (4 %) tibia fractures in children aged >1 year were reported for abuse, which is in agreement with previous studies.

Corner fractures are reported to be highly specific for abuse during the first year of life [4, 6, 12, 32]. Corner fractures result from planar fractures through the primary spongiosa, resulting in multiple microfractures across the metaphysis. These microfractures occur in unmineralized bone almost exclusively in those aged <2 years [6]. In the current study, six patients aged 1–4 months who were reported for abuse had femoral corner fractures and another six patients aged 1–4 months who were reported for abuse had tibial corner fractures. Therefore, corner fractures did not occur in children aged >1 year. All children with corner fractures presented with multiple other fractures. Corner fractures are a highly specific indicator for abuse [4, 32].

We report the highest percentage of abuse in African American (43 %), unknown (29 %), and Hispanic patients (25 %), followed by White (12 %) and other (12 %). Prior studies have indicated that White and African American races are reported most frequently for child abuse, although the actual demographics of reporting seem to vary by region [7, 9]. Both of these meta-analyses found more absolute cases of child abuse in Whites but a higher relative risk associated with being African American. In the current study, 29 % of the reported races were unknown, providing a potential reason as to why our data on reported abuse differs from some studies looking at abuse demographics [7, 9, 14]. This finding reinforces the need for more accurate reporting of demographics to appropriately assess relative and absolute risk of demographics in non-accidental fractures, as mentioned by other authors [9].

The inequality of reporting by insurance type noted in our study (7 % of patients with commercial insurance and fractures were reported vs 28 % of those with Medicaid and fractures) is consistent with previous studies [7, 9, 14]. This may represent under-reporting of children with commercial insurance, over-reporting of children with Medicaid, or it may accurately represent the epidemiology of child abuse. In the current study, 15 of 19 (79 %) children reported for abuse were insured by Medicaid. In the same 19 cases, 16 (84 %) presented with multiple fractures and inconsistent histories, which suggests our reporting is consistent with clinical presentation independent of insurance status.

About one in ten cases of reported child abuse are substantiated in the United States [21, 33]. The data on abuse includes all types of maltreatment including sexual abuse, neglect, non-fracture physical abuse, and fractures. Despite the predominance of unsubstantiated cases, some studies suggest fractures are not being reported accurately due to charting deficiencies [34–36]. We did not evaluate charting deficiencies in the current study. We agree that more detailed charting may decrease the number of falsely reported cases and decrease the number of missed cases that should have been reported.

Limitations of the study include the retrospective nature and the lack of data regarding substantiation by the Pennsylvania Office of Children, Youth, and Families. However, in 16 of 19 cases the child sustained multiple fractures that strongly indicate abuse. In only three cases did children sustain isolated fractures which may be determined to be secondary to an accident.

In summary, fractures in children aged <1 year are more commonly reported as NAT (32 %) as opposed to those aged 1−2 years (5 %). Of those reported as NAT, the most predictive factor for reporting were multiple fractures. Thirty-four percent of patients with multiple fractures were reported for abuse, which constituted 84 % of all cases reported for abuse in this study. Eight of the 11 femoral fractures reported for abuse were transverse fractures while only three were spiral fractures. One hundred percent of corner fractures were reported for abuse, none of which occurred in isolation. Most (70 %) patients reported for abuse also presented to the ED as opposed to clinic.
