
JOURNAL INFORMATION
==============================
NLM Title Abbreviation: J Child Orthop
Journal ID: J Child Orthop
NLM Journal ID: 101313582

Journal of Children's Orthopaedics

ISSN: 1863-2521
EISSN: 1863-2548
Publisher: SAGE Publications

ARTICLE INFORMATION
==============================
PMCID: PMC5033783
PMID: 27538941
DOI: 10.1007/s11832-016-0764-2
Article ID: 764
Article version: 1
Subjects: Erratum

Erratum to: Identifying non-accidental fractures in children aged <2 years

Leaman Laura A. 2
Hennrikus William L. 1
http://orcid.org/0000-0002-9712-4629 Bresnahan James J. +1-717-531-7066 jbresnahan@hmc.psu.edu
1 3
1 Department of Orthopaedics and Rehabilitation, The Pennsylvania State University College of Medicine, Hershey, PA USA
2 Department of Family Medicine, Lancaster General Health, Lancaster, PA USA
3 Pennsylvania State University College of Medicine, 500 University Dr., Hershey, PA USA
Electronic publication date: 2016 Aug 18
Print publication date: 2016 Oct
Volume: 10
Issue: 5
First page: 467
Last page: 467
Copyright: © The Author(s) 2016
License: This article is distributed under the terms of the Creative Commons Attribution 4.0 International License (http://creativecommons.org/licenses/by/4.0/), which permits unrestricted use, distribution, and reproduction in any medium, provided you give appropriate credit to the original author(s) and the source, provide a link to the Creative Commons license, and indicate if changes were made.
License URL: https://creativecommons.org/licenses/by/4.0/

Erratum for: Identifying non-accidental fractures in children aged <2 years 10 4 23 6 2016 335 341 Journal of Children's Orthopaedics 10.1007/s11832-016-0755-3 PMC4940250 27339476
issue-copyright-statement: © The Author(s) 2016

==============================
Open Access

This article is distributed under the terms of the Creative Commons Attribution 4.0 International License (http://creativecommons.org/licenses/by/4.0/), which permits unrestricted use, distribution, and reproduction in any medium, provided you give appropriate credit to the original author(s) and the source, provide a link to the Creative Commons license, and indicate if changes were made.

The online version of the original article can be found under doi:10.1007/s11832-016-0755-3.

